# Neuro-Behçet’s Disease Onset in the Context of Tuberculous Meningoencephalitis: A Case Report

**DOI:** 10.3390/medicina59122163

**Published:** 2023-12-13

**Authors:** Florian Antonescu, Ioana Butnariu, Dana Antonescu-Ghelmez, Sorin Tuta, Bianca Adriana Voinescu, Mihnea Costin Manea, Amanda Ioana Bucur, Altay Sercan Chelmambet, Adriana Moraru

**Affiliations:** 1 Department of Clinical Neurosciences, “Carol Davila” University of Medicine and Pharmacy, 020023 Bucharest, Romaniamihnea.manea@umfcd.ro (M.C.M.); 2 Department of Neurology, National Institute of Neurology and Neurovascular Diseases, 041915 Bucharest, Romania; 3 “Victor Babes” Clinical Hospital for Infectious and Tropical Diseases, 030303 Bucharest, Romania; 4 “Prof. Dr. Alexandru Obregia” Clinical Psychiatry Hospital, 041915 Bucharest, Romania

**Keywords:** Behçet’s disease, Neuro-Behcet’s disease, tuberculous meningoencephalitis, systemic vasculitis, HLA B51 screening

## Abstract

Behçet’s disease (BD) is a systemic vasculitis that frequently presents with a relapsing–remitting pattern. CNS involvement (Neuro-Behçet) is rare, affecting approximately 10% of patients. Its etiological mechanisms are not yet fully understood. The most commonly accepted hypothesis is that of a systemic inflammatory reaction triggered by an infectious agent or by an autoantigen, such as heat shock protein, in genetically predisposed individuals. Mycobacterium tuberculosis is known to be closely interconnected with BD, both affecting cell-mediated immunity to a certain extent and probably sharing a common genetic background. We present the case of a 34-year-old Caucasian woman who had been diagnosed with tuberculous meningitis 15 months prior, with significant neurological deficits and lesional burden on MRI with repeated relapses whenever treatment withdrawal was attempted. These relapses were initially considered as reactivation of tuberculous meningoencephalitis, and symptoms improved after a combination of antituberculous treatment and corticosteroid therapy. After the second relapse, the diagnosis was reconsidered, as new information emerged about oral and genital aphthous lesions, making us suspect a BD diagnosis. HLA B51 testing was positive, antituberculous treatment was stopped, and the patient was started on high doses of oral Cortisone and Azathioprine. Consequently, the evolution was favorable, with no further relapses and slow improvements in neurological deficits. To our knowledge, this is the first report of Neuro-Behçet’s disease onset precipitated by tuberculous meningitis. We include a review of the available literature on this subject. Our case reinforces the fact that Mycobacterium tuberculosis infection can precipitate BD in genetically predisposed patients, and we recommend HLA B51 screening in patients with prolonged or relapsing meningoencephalitis, even if an infectious agent is apparently involved.

## 1. Introduction

Behçet’s disease (BD) is a systemic vasculitis, frequently presenting with a recurrent–relapsing pattern, involving both arterial and venous vessels of different calibers. Typically, BD presents with recurrent oral aphthae, followed by eye and joint involvement and genital ulcers [1]. 

Neurological involvement is rare, averaging just under 10%, and both the central nervous system and the peripheral nervous system can be affected [2]. A large retrospective study of 387 patients diagnosed with BD over a 20-year period in Turkey found a frequency of Neuro-Beçhet’s disease (NBD) of 13% in men and 5.6% in women [3]. Many other reports consistently support a gender disparity, with NBD affecting men 2.8-times more often than women, with the average age of onset in the third decade [2,4]. Extension to the central nervous system can embrace many forms of presentation: migraine-like attacks, cerebral venous thrombosis, diffuse cerebral parenchymal involvement, and neuro-psycho Behçet’s syndrome [5]. 

Although its etiopathogenesis has not yet been fully understood, multiple theories have been set forth, one of the most accepted being that of a systemic inflammatory reaction triggered recurrently by infectious agents, such as herpes simplex virus (HSV)-1 and Streptococcus sanguinis, or by an autoantigen, such as heat shock proteins, in genetically predisposed individuals [6,7]. 

The relationship between Mycobacterium tuberculosis (MT) and BD is complex and not entirely clarified. On the one hand, the cell-mediated immunity defects produced by BD increase a patient’s susceptibility to tuberculosis (TB); on the other hand, TB seems to increase the risk and severity of BD [8,9].

## 2. Case Presentation

**Figure 1:**
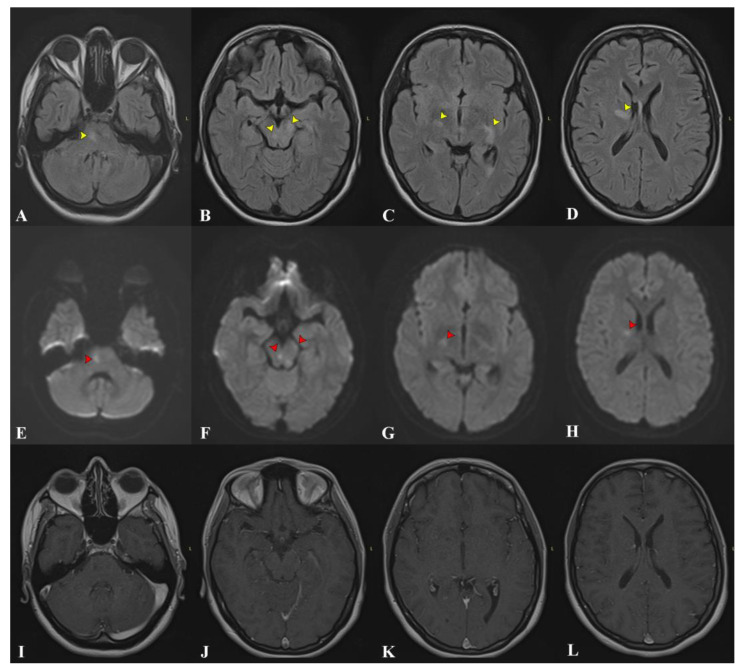
The first cerebral MRI examination at the onset of symptoms. (**A**–**D**) FLAIR images reveal disseminated infra- and supratentorial hyperintense lesions (yellow arrows). (**E**–**H**) DWI images show corresponding restriction of diffusion (red arrows) for the majority of the lesions visible on FLAIR. (**I**–**L**) Contrast-enhanced corresponding sections without any pathological enhancement.

We present the case of a 34-year-old Caucasian woman without prior medical history who had an acute onset with low fever, headache, and persistent dry cough. As the symptoms persisted unabated for about a week, she was referred and admitted to an infectious disease clinic. She was already under surveillance as, just a few weeks prior, her husband and one of her work colleagues had been diagnosed with active pulmonary TB. About a week after admission, she developed speech and walking difficulties. A cerebral MRI examination was performed, revealing multiple T2/FLAIR hyperintense areas, most of them with restricted diffusion without gadolinium enhancement. These areas were distributed bilaterally in the brainstem, the periventricular white matter, and the right internal capsule, suggestive of inflammatory lesions (Figure 1). A lumbar puncture was performed, showing pleocytosis (>500 elements/uL), increased proteinorrachia with significant hyperalbuminorrachia, and a positive PCR GeneXpert for MT. CSF cultures, which arrived later, were also positive. The patient tested negative for HIV.

Given the recent exposure, the presence of systemic inflammation, neurological deficits, and a positive PCR CSF analysis for MT, a diagnosis of tuberculous meningoencephalitis was made, and the patient was started on tuberculostatic therapy consisting of Isoniazid, Rifampicin, Ethambutol, and Pyrazinamide in association with intravenous Dexamethasone. The evolution was favorable, with remission of the systemic inflammatory signs, marked improvement in gait and dysarthria, and normalization of CSF parameters.

**Figure 2:**
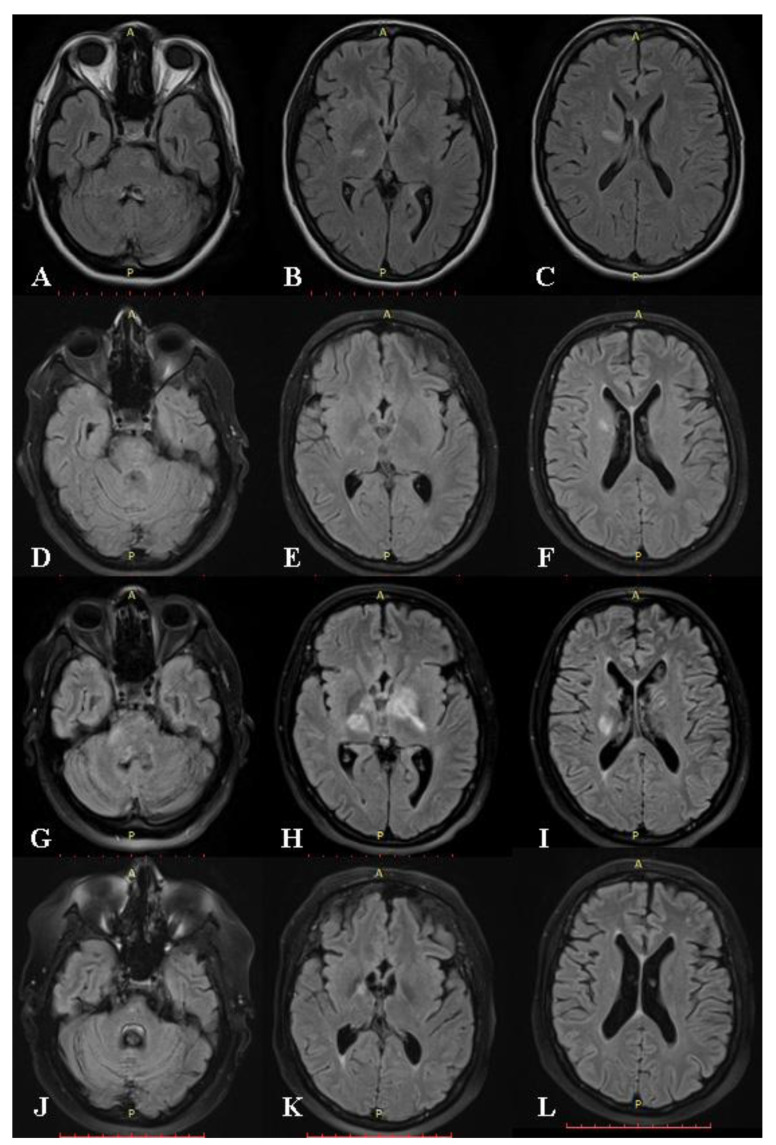
Corresponding FLAIR images at different moments in time. (**A**–**C**) The first MRI examination, the same examination that is presented in Figure 1, with lesions affecting the brain stem, left capsule, and bilateral periventricular white matter. (**D**–**F**) MRI scan at 12 months from onset showing favorable evolution, with significant regression of lesions. (**G**–**I**) MRI scan 9 months later, during a severe relapse, with new pontine lesions and bilateral thalamic lesions extending to the left basal ganglia and the right periventricular white matter. Significant atrophy is visible comparatively with the previous examinations. (**J**–**L**) MRI scan at 3 years from onset showing, once again, lesion regression under treatment. Marked progression of atrophy is visible. A—anterior and P—posterior.

At the one-year follow-up, worsening of the gait disorder was noted, with added dysphonia and a slight cognitive impairment. At that moment, she was undergoing tapering of the tuberculostatic medication and was following a 3/7 scheme. A cerebral MRI scan showed the resolution of the majority of known lesions without any new ones, remission of restriction of diffusion, and still no pathological enhancement (Figure 2D–F). The symptoms were considered a relapse of the TB meningoencephalitis, precipitated by medication tapering, and treatment was escalated to a 7/7 scheme, with added corticotherapy for the first 3 weeks. She presented partial improvement in the neurological deficits without significant adverse effects. It was at this moment, due to the persistence of neurological deficits and the ongoing SARS-CoV-2 pandemic, which had clogged the infectious disease clinics, that the patient was referred to our medical facility for follow-up.

At admission to our clinic, 15 months after the initial diagnosis, the clinical examination revealed a slight ataxia of the right limbs and mild dysarthria without any motor deficits. Cognitive testing showed attention deficits and a significant reduction in processing speed. A lumbar puncture showed slight pleocytosis (35 elements/uL), a negative Ziehl Nielsen smear, and a negative GeneXpert for MT. MRI was not significantly modified from the previous examination. Even though TB meningoencephalitis does not usually have a relapsing pattern, as the patient had improved under treatment, we found little reason to challenge the diagnosis. Still, the negative GeneXpert should have raised our suspicion. 

**Figure 3:**
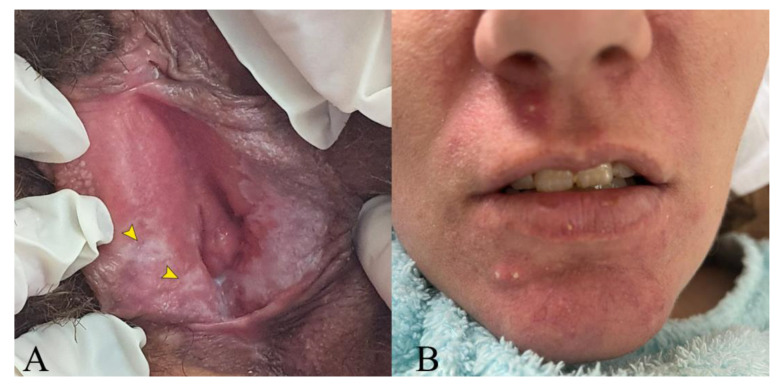
(**A**) Genital scarring from previous ulcerations on the right labia minora (yellow arrows). (**B**) Facial acne lesions, which had a tendency to disappear under corticotherapy.

The patient’s clinical and neurological condition remained stationary for the next 6 months under the escalated treatment scheme, and then, her neurological deficits abruptly worsened once again, this time under correctly conducted tuberculostatic treatment. The neurological exam revealed moderate dysarthria, astazoabasia, and right ataxic hemiparesis with a positive right Babinski sign. The cognitive decline had progressed significantly, with prominent slowing, obvious memory impairment, and pseudobulbar affect. An MRI showed new brainstem and bilateral thalamic lesions extending to the basal ganglia and the periventricular white matter. Global atrophy was becoming evident (Figure 2G–I). She was once again hospitalized, and the diagnosis of tuberculous meningoencephalitis was reconsidered. A detailed history with targeted questions revealed recurrent oral and genital ulcers and perioral acneiform lesions, which had first appeared at the same time as the original symptoms and were alleviated periodically when corticosteroids were introduced. Indeed, a genital examination revealed cicatricial lesions on the labia minora from prior painful ulcerations (Figure 3). We observed no oral mucosal lesions, but the patient’s husband confirmed a history of repeating oral ulcers that sometimes caused enough discomfort to interfere with feeding. Blood tests were negative for vasculitis and autoimmune demyelinating diseases. CSF tested negative for neuroborreliosis, neurosyphilis, HSV-1, and HSV-2. CSF MT GeneXpert was repeated once again and was negative. Considering sarcoidosis, a thoracic CT scan was performed, but there were no pathological findings. Given the recurrent relapsing pattern and mucocutaneous oral and genital ulcers, the patient was tested for HLA B51, and the results came back positive. At this moment, she scored 6 points on the International Criteria for Behçet’s Disease [10]. Antituberculous treatment was stopped, and the patient was started on corticotherapy, having been discharged with Prednisone 1 mg/kg/day with a favorable response. Later, as part of corticoid-sparing treatment, she was started on Azathioprine (AZA), gradually reaching a dose of 150 mg per day, after which corticosteroids were progressively withdrawn. Now, about 3 years from the onset, the patient is stable, with some residual neurological deficits and moderate cognitive decline but no clinical or MRI relapses (Figure 2J–L).

## 3. Discussion

Neurological involvement in BD (NBD) can be classified into two major forms: parenchymal NBD, or intra-axial NBD, and non-parenchymal, also known as extra-axial or vascular NBD [4]. Parenchymal NBD is encountered more frequently, representing 70–80% of cases, and can take on multiple clinical forms, such as brainstem, hemispheric, spinal, or meningoencephalitic syndromes [11]. Vascular NBD presents mostly as thrombosis of the cerebral venous sinuses and is usually associated with a better prognosis. Arterial involvement is rare and usually affects large intracranial arteries [12]. 

NBD usually has a relapse–remitting pattern, and the presence of HLA B51 increases the risk of another relapse by 3.6-times, according to Noel et al. [13]. Other known factors associated with a poorer prognosis are parenchymal involvement, increased protein and cell numbers in CSF, extensive lesion involvement, and spinal involvement [3]. In addition to the relapsing pattern, NBD may present with a chronic progressive pattern, mimicking progressive multiple sclerosis [14]. This pattern is strongly associated with increased levels of IL-6 in CSF and a significant progression of cerebral atrophy, even in the absence of new focal lesions on the MRI [14,15,16].

Psychiatric involvement may also be a feature of NBD, with memory impairment being the most common finding [17]. Cognitive decline can affect individuals with BD, even in the absence of overt NBD, and seems to be able to progress independently of the CNS focal structural abnormalities, paralleling the progression of atrophy [12,18]. 

### 3.1. Relationship between Behçet Disease and Tuberculosis

The relationship between BD and MT has long been thought to be bimodal. MT has the potential to elicit BD through complex mechanisms, including cross-immunoreactivity against host heat shock protein (HSP) 60, and to sustain the production of various pro-inflammatory cytokines, such as TNF, IL-6, and IL-8 [19]. The physiopathological mechanisms are yet unclear; still, Th1 and Th17 are involved in both pathologies and could represent a common pathway [20]. This does not come as a surprise, as TB is known to interfere with the immune system and the regulation of the mechanism of inflammation and can trigger rheumatologic syndromes or worsen pre-existent rheumatoid pathology [20,21].

The interference extends to the genetic level, as a recent study has shown that gene configurations that increase the risk of TB are also associated with an increased risk of developing Behçet disease. Despite some limitations, additional data in the same study suggest that past or present TB infection may be an independent risk factor for BD [8]. 

MT has known interactions with the broad antigen HLA B5: carriers of the HLA B51 variant have a higher susceptibility for developing TB, while HLA-B52-positive individuals were proven to be less prone to pulmonary damage in Mycobacterium infections [22]. Moreover, in a recent study by Shen et al., BD patients with latent Mycobacterium infection were shown to have worse clinical outcomes, with more severe dermatological and ophthalmological damage [20]. Moreover, BD patients with active TB seem more prone to having systemic symptoms, including fever and weight loss, arthritis, and thrombotic events [23].

Conversely, BD may render individuals more vulnerable to TB infection due to an impaired immune system [20]. Furthermore, corticotherapy and immunosuppressants used to treat BD can reactivate a latent mycobacterium infection or facilitate a new one. This is especially true in the case of TNF-α, inhibitors, which are known to favor TB infection by disrupting granuloma formation, with the greatest risk observed in the first 28 weeks of treatment [24,25,26].

### 3.2. Approaching a Difficult Differential Diagnosis

The clinical diagnosis of BD can be a challenge since there are currently no direct tests that can confirm or exclude it. Over the years, multiple diagnostic criteria have been proposed, the most recent being the 2013 International Criteria for Behçet’s Disease, briefly the ICBD criteria, which were shown to have a sensitivity of close to 95% and a specificity of 90% [10,27,28]. The numbers are encouraging, but the diagnosis is still not straightforward. Oral and genital ulcers are the pillars of BD diagnosis, but they may be absent at the moment of the clinical examination, and patients often fail to report them in their history.

The criteria are based on clinical extensions of the disease to different organs. This can be confounding, especially when confronted with diseases that can also involve all these organs, such as extrapulmonary TB. Pseudo-Behçet’s disease is an umbrella term for clinical presentations with oral and genital ulcerations that, in the end, are proven to have other etiologies. This has been reported in various dermatological and rheumatological conditions but also in TB patients [29].

TB meningitis affects probably under 1% of TB patients, but this number can be significant in areas where the disease is rife [30]. Considering the fact that Romania has the highest TB burden in the EU, we can personally attest to this [31]. Acute aseptic meningitis is a known form of presentation of NBD and usually responds well to corticotherapy [32,33]. As in our case, this can prove to be a confounding factor, as corticosteroids are also routinely used in the treatment of infectious meningitis.

TB patients can present with skin-aseptic nodular lesions called tuberculids, which can also involve genital mucosae [34]. Oral ulcers in TB are rare, affecting under 1% of patients, but their incidence seems to be on the rise [35]. The difference between TB pseudo-Behçet’s disease and BD is the clinical response to treatment, with symptoms remitting completely under tuberculostatic treatment in the first case and needing immunosuppressive therapy in the second [9,29].

One of the peculiarities of our case is represented by the simultaneous onset of TB meningitis and NBD. Although the GeneXpert and CSF cultures are indispensable in diagnosing pulmonary or extrapulmonary TB, it must be highlighted that even these highly sensitive and specific laboratory tests can leave room for doubt. At the moment, the mycobacterial culture represents the gold standard for the diagnosis of TB, and according to the World Health Organization (WHO), it has to be tested in every clinical suspicion of tuberculous meningitis [36]. Studies have demonstrated that nucleic acid amplification tests (NAATs) demonstrate superior performance compared with the microbiological diagnosis, with a pooled sensitivity and specificity of 96% and 92%, respectively [37]. However, those values decrease significantly when non-respiratory specimens are taken into account. A meta-analysis showed sensitivity and specificity values of 70% and 97%, respectively [38]. 

## 4. Conclusions

BD can present as meningoencephalitis by itself but can also accompany or be triggered by TB meningoencephalitis. To our knowledge, our case is the first report of the simultaneous onset of tuberculous meningoencephalitis and NBD, and we found that this type of presentation raises particular issues.

Differentiating between the two is not easy and can prove, as in our case, practically impossible in the early stages. Orogenital syndrome is highly suggestive of BD but is not sufficient in such cases, as shown by reported cases of TB pseudo-Behçet’s disease. HLA B51 and CSF IL-6 testing are very useful in orienting the diagnosis. Close monitoring and the attempt to differentiate between treatment response to anti-infectious medication and corticotherapy seem to be the key. The two drug regimens should be tapered separately, and the patient should be closely monitored for relapses or insidious progression. We think that this should include periodic cognitive testing. If possible, DB patients should be tested for latent TB.

## Data Availability

Data are contained within the article. No new data were created or analyzed in this study. Data sharing is not applicable to this article.
